# Risk factors for flare and treatment of disease flares during pregnancy in rheumatoid arthritis and axial spondyloarthritis patients

**DOI:** 10.1186/s13075-017-1269-1

**Published:** 2017-03-20

**Authors:** Stephanie van den Brandt, Astrid Zbinden, Dominique Baeten, Peter M. Villiger, Monika Østensen, Frauke Förger

**Affiliations:** 1 Department of Rheumatology, Immunology and Allergology, University Hospital, University of Bern, Freiburgstrasse 8, 3010 Bern, Switzerland; 2 Clinical Immunology and Rheumatology, Academic Medical Center/University of Amsterdam, Meibergdreef 9, 1105 AZ Amsterdam, The Netherlands

**Keywords:** Rheumatoid arthritis, Axial spondyloarthritis, Tumor necrosis factor inhibitors, Treatment, Pregnancy

## Abstract

**Background:**

During pregnancy, patients with rheumatoid arthritis (RA) and axial spondyloarthritis (axSpA) can experience active disease, which might be influenced by adjustment of treatment around conception. The aim of this study was to identify possible risk factors of disease flares during pregnancy and to evaluate the effect of treatment in pregnant patients experiencing a flare.

**Methods:**

Pregnant patients with RA and axSpA were prospectively followed before, during, and after pregnancy. Disease activity and flares of disease activity were analyzed in regard to medication.

**Results:**

Among 136 pregnant patients, disease flares during pregnancy occurred in 29% of patients with RA and in 25% of patients with axSpA. In both diseases, active disease and tumor necrosis factor inhibitor (TNFi) discontinuation in early pregnancy were identified as risk factors for disease flares during pregnancy. Of 75 patients with RA, 15 patients were on TNFi and discontinued the treatment at the time of the positive pregnancy test. After stopping TNFi, disease activity increased, which was reflected by peaking C-reactive protein levels at the first trimester. The relative risk of flare in patients with RA stopping TNFi was 3.33 (95% CI 1.8–6.1). Initiation of TNFi or glucocorticosteroid (GC) treatment in 60% of these patients resulted in disease improvement at the second and third trimesters. In comparison, patients with RA without TNFi in the preconception period, most of whom had used pregnancy-compatible antirheumatic drugs, showed mild and stable disease activity before and during pregnancy. Of 61 patients with axSpA, 24 patients were on TNFi and discontinued the treatment at the time of the positive pregnancy test. In patients with axSpA stopping TNFi, a disease aggravation at the second trimester could be observed. The relative risk of flare in this group was 3.08 (95% CI 1.2–7.9). In spite of initiated TNFi or GC treatment in 62.5% of these patients, disease activity remained elevated throughout pregnancy. Patients with axSpA without TNFi in the preconception period showed persistent high disease activity from prepregnancy until the postpartum period.

**Conclusions:**

On the basis of a risk-benefit analysis, to stabilize disease activity and to prevent a flare during pregnancy in patients with RA and axSpA, tailored medication including TNF inhibitors should be considered beyond conception.

**Electronic supplementary material:**

The online version of this article (doi:10.1186/s13075-017-1269-1) contains supplementary material, which is available to authorized users.

## Background

Rheumatoid arthritis (RA) and axial spondyloarthritis (axSpA) often affect women of childbearing age. In patients wishing for children, pregnancy represents a challenge in the management of the disease because current disease activity, the influence of pregnancy on the disease, and the safety of antirheumatic drugs in pregnancy have to be taken into account [1]. During pregnancy, active disease can be found in about 35% to 52% of patients with RA and in 60% to 80% of patients with axSpA [2–5].

In RA, the positivity for or elevated level of rheumatoid factor or anticitrullinated antibodies is associated with more active disease during pregnancy [6, 7]. Disease activity before conception seems to be an additional factor that influences the disease course during pregnancy in patients with RA because quiescent disease often remains stable throughout pregnancy [3]. Active disease or flares during pregnancy in patients with rheumatic disease can harm maternal and fetal health and should therefore be avoided.

Up to now, it is unknown whether changes of treatment before conception or early in pregnancy have an influence on the disease course during pregnancy in patients with RA and axSpA. This information would be of clinical importance, however, in the management of patients with RA and axSpA before and during pregnancy.

The aim of this study was to analyze the frequency of flares during pregnancy in patients with RA and axSpA and to delineate risk factors for flares. In addition, we analyzed the response to therapy in controlling disease activity after the occurrence of a flare in both diseases during pregnancy.

## Methods

### Patients and medications

In total, 136 pregnant patients with RA or axSpA were prospectively followed between 2000 and 2015 at the Center for Pregnancy in Rheumatic Diseases at the Department of Rheumatology of the Inselspital Bern, Switzerland. Patients with RA had to fulfill the revised 1987 American College of Rheumatology classification criteria for RA [8]. Patients with spondyloarthritis (SpA) fulfilled the European Spondylarthropathy Study Group criteria for SpA [9]. All patients with SpA also fulfilled the Assessment of SpondyloArthritis international Society (ASAS) criteria for axSpA; for patients recruited before 2009, the ASAS criteria were applied in retrospect [10]. The study was approved by the ethics committee of the Canton of Bern, Switzerland, and patients were included after they provided written informed consent. Patients were examined within 6 months before conception, at each trimester (gestational weeks 10–12, 20–22, and 30–32) and 6–8 weeks postpartum. At each visit, detailed information on medication, disease activity, and pregnancy were recorded. Flare was analyzed at each visit during pregnancy and postpartum. If demanded by maternal disease, tumor necrosis factor inhibitor (TNFi) or glucocorticosteroid (GC) treatment was initiated during pregnancy, based on a risk-benefit analysis as well as informed and shared decision making.

### Assessments and definitions

In patients with RA, disease activity states were assessed by the three-variable Disease Activity Score in 28 joints based on C-reactive protein (DAS28-CRP) [11]. DAS28-CRP scores below 2.6 were defined as clinical remission, DAS28-CRP scores from 2.7 to 3.2 as low disease activity, DAS28-CRP scores from 3.3 to 5.1 as intermediate disease activity, and DAS28-CRP scores above 5.1 as high disease activity [11]. Flare was defined by an increase of DAS28-CRP greater than 0.6 [12] in combination with an elevated C-reactive protein (CRP) level. Active disease was defined by DAS28-CRP scores higher than 3.2. In patients with axSpA, disease activity states were measured by the patient global assessment, the Bath Ankylosing Spondylitis Disease Activity Index, and the Ankylosing Spondylitis Disease Activity Score based on C-reactive protein (ASDAS-CRP). In patients with axSpA recruited before the development of the ASDAS-CRP in 2009 [13], the ASDAS-CRP scores were calculated retrospectively. Categories of the ASDAS-CRP were defined as inactive disease for scores below 1.3, moderate disease activity for scores from 1.3 to 2.0, high disease activity for scores from 2.1 to 3.5, and very high disease activity for scores above 3.5 [14]. Active disease was defined by ASDAS-CRP scores higher than 2.1. Flare of disease activity was defined by an increase of ASDAS-CRP greater than 0.6 in combination with an elevated CRP level. A flare during pregnancy was defined as a flare observed during the first, second, or third trimester.

### Statistical analysis

For longitudinal comparisons of paired samples, the Wilcoxon signed-rank test was used. The Mann-Whitney *U* test was performed to analyze unpaired data as well as in groupwise comparisons. To analyze categorical data, Fisher’s exact test was performed. A significant difference was considered in case of *P* values less than 0.05.

## Results

### Flare rates during pregnancy in patients with RA and axSpA are associated with active disease and TNFi discontinuation in early pregnancy

A total of 136 pregnant patients were identified, comprising 75 patients with RA and 61 patients with axSpA. Patients’ characteristics and medical treatment at baseline are displayed in Table 1.

**Table 1:** Patients’ characteristics and treatments before conception

	RA (*n* = 75)	axSpA (*n* = 61)
Age at conception, years (median)	31 (21–40)	31 (22–41)
Age at diagnosis, years (median)	27 (20–38)	24 (17–33)
Rheumatoid factor-positive	50 (66)	–
HLA-B27-positive	–	43 (70.5)
axSpA with peripheral arthritis	–	27 (44.3)
Medication within 3 months before conception		
Methotrexate^a^	2 (2.7)	–
NSAIDs	8 (10.7)	18 (29.5)
TNFi^b^	15 (20.0)	24 (39.3)
Glucocorticosteroids^c^	25 (33.3)	5 (8.2)
DMARDs	29 (38.7)	5 (8.2)
SSZ	18 (24.0)	5 (8.2)
HCQ	8 (10.7)	
SSZ and HCQ	3 (4.0)	

*Abbreviations: axSpA* Axial spondyloarthritis, *DMARD* Disease-modifying antirheumatic drug, *HCQ* Hydrochloroquine, *HLA* Human leukocyte antigen, *NSAID* Nonsteroidal anti-inflammatory drug, *RA* Rheumatoid arthritis, *SSZ* Sulfasalazine, *TNFi* Tumor necrosis factor inhibitor

^a^Methotrexate, discontinued 1 month before the planned conception

^b^TNFi, discontinued at the time of the positive pregnancy test

^c^Prednisone or prednisolone

Before pregnancy, 61 patients with RA had low disease activity, and 8.6% had active disease with DAS28-CRP scores greater than or equal to 3.2. However, during pregnancy, a flare of disease activity occurred in 29% of patients with RA. Most flares emerged in the first trimester (Table 2). No patient with RA experienced more than one episode of flare during pregnancy. Comparing patients with flares with those without them, the discontinuation of TNFi in early pregnancy correlated with the risk of flares (*P* = 0.001) (Table 3). Flares during the course of pregnancy were also associated with elevated disease activity and CRP at early pregnancy (*P* = 0.038 and *P* = 0.008, respectively) (Table 3 and Additional file 1: Table S1).

**Table 2:** Flares and medical treatments during pregnancy in patients with and without tumor necrosis factor inhibitor use until early pregnancy

	RA		axSpA	
	TNFi until positive pregnancy test^a^ (*n* = 15)	TNFi-negative (*n* = 60)	TNFi until positive pregnancy test (*n* = 24)	TNFi-negative (*n* = 37)
Patients (n) with flare at				
First trimester	6	4	1	2
Second trimester	3	4	5	1
Third trimester	1	4	4	2
Medication at conception				
TNFi	15 (100)	0 (0)	24 (100)	0 (0)
NSAIDs	1 (6.7)	13 (21.7)	11 (45.8)	23 (61.2)
Glucocorticosteroids^b^	1 (6.7)	23 (38.3)	2 (8.3)	1(3.7)
DMARDs	2 (13.3)	27 (45)	3 (12.5)	2 (5.4)
SSZ	1 (6.7)	17(28.3)	3 (12.5)	2 (5.4)
HCQ	1 (6.7)	7 (11.7)		
SSZ and HCQ		3 (5)		
Medication during pregnancy				
TNFi	4 (26.7)	5 (8.3)	10 (41.7)	1 (2.7)
NSAIDs	5 (33.3)	23 (38.3)	15 (62.5)	27 (73)
Glucocorticosteroids^b^	7 (46.7)	29 (48.3)	12 (50.0)	3 (8.1)
DMARDs	7 (46.7)	38 (63.3)	3 (12.5)	2 (5.4)
SSZ	1 (6.7)	23 (38.3)	3 (12.5)	2 (5.4)
HCQ	1 (6.7)	9 (15)		
SSZ and HCQ	5 (33.3)	6 (10)		
Initiation of medication during pregnancy	9 (60)	16 (26.7)	15 (62.5)	4 (10.8)
TNFi^c^	4 (26.7)	5 (8.3)	10 (41.7)	1 (2.7)
Start at visit 1 T-2 T-3 T	2-2-0	1-3-1	4-4-2	0-0-1
Glucocorticosteroids^b^	5 (33.3)	11(18.6)	12 (50)	3 (8.1)
Start at visit 1 T-2 T-3 T	0-4-1	3-6-2	4-5-3	0-2-1

*Abbreviations: axSpA* Axial spondyloarthritis, *DMARD* Disease-modifying antirheumatic drug, *HCQ* Hydrochloroquine, *NSAID* Nonsteroidal anti-inflammatory drug, *RA* Rheumatoid arthritis, *SSZ* Sulfasalazine, *TNFi* Tumor necrosis factor inhibitor, *1 T* First trimester, *2 T* Second trimester, *3 T* Third trimester

Numbers are count or count (percent); the percentages are calculated for each column

^a^NSAIDs used until gestational week 32

^b^Prednisone or prednisolone

^c^TNFi initiated during pregnancy: 11 certolizumab, 8 etanercept, 1 adalimumab

**Table 3:** Risk factors for flare during the course of pregnancy

	Patients with RA (*n* = 75)					Patients with axSpA (*n* = 61)				
	Flare	No flare	RR	95% CI	*P* value	Flare	No flare	RR	95% CI	*P* value
Treatment										
TNFi before pregnancy^a^ and discontinued at positive pregnancy test	10 of 22 (45.5)	5 of 53 (9.4)	3.333	(1.8–6.1)	0.001*	10 of 15 (66.7)	14 of 46 (30.4)	3.083	(1.2–7.9)	0.017*
TNFi during first trimester	3 of 22 (13.6)	5 of 53 (9.4)	1.322	(0.5–3.5)	0.686	6 of 15 (40)	12 of 46 (26.1)	1.593	(0.7–3.8)	0.340
GCs before pregnancy	8 of 22 (36.4)	17 of 53 (32.1)	1.143	(0.6–2.4)	0.790	1 of 15 (6.7)	4 of 46 (8.7)	0.800	(0.1–4.9)	0.642
GCs during first trimester	8 of 22 (36.4)	17 of 53 (32.1)	1.143	(0.6–2.4)	0.790	1 of 15 (6.7)	6 of 46 (13)	0.551	(0.1–3.6)	0.178
DMARDs before pregnancy	10 of 22 (45.5)	22 of 53 (41.5)	1.120	(0.6–2.3)	0.801	3 of 15 (20)	5 of 46 (10.9)	1.656	(0.6–4.6)	0.589
DMARDs during first trimester	8 of 22 (36.4)	22 of 53 (42.5)	0.860	(0.4–1.8)	0.798	2 of 15 (13.3)	3 of 46 (6.5)	1.723	(0.5–5.6)	0.589
NSAIDs before pregnancy	2 of 22 (9.1)	6 of 53 (11.3)	0.838	(0.2–2.9)	0.568	3 of 15 (20)	15 of 46 (32.6)	0.597	(0.2–1.9)	0.518
NSAIDs during first trimester	3 of 22 (13.6)	6 of 53 (11.3)	1.158	(0.4–3.1)	0.716	3 of 15 (20)	15 of 46 (32.6)	0.597	(0.2–1.9)	0.518
Disease activity										
Active disease before pregnancy^a^	5 of 22 (22.7)	9 of 53 (17.0)	1.282	(0.6–2.9)	0.536	2 of 15 (13.3)	4 of 46 (8.7)	1.410	(0.4–4.8)	0.630
Active disease during first trimester	8 of 22 (36.4)	10 of 53 (18.9)	0.456	(0.2–0.9)	0.038*	5 of 15 (33.3)	11 of 46 (23.9)	0.717	(0.3–1.7)	0.510
Elevated CRP before pregnancy^a^	3 of 22 (13.6)	8 of 53 (15.1)	0.584	(0.3–1.3)	0.282	1 of 15 (6.7)	2 of 46 (4.3)	0.724	(0.1–3.8)	0.718
Elevated CRP during first trimester	9 of 22 (40.9)	12 of 53 (22.6)	0.553	(0.2–0.7)	0.008*	7 of 15 (46.7)	6 of 46 (13)	0.280	(0.1–0.7)	0.006*

*Abbreviations: axSpA* Axial spondyloarthritis, *CRP* C-reactive protein, *DMARD* Disease-modifying antirheumatic drug, *GC* Glucocorticosteroid, *NSAID* Nonsteroidal anti-inflammatory drug, *RA* Rheumatoid arthritis, *RR* Relative risk, *SpA* Spondyloarthritis, *TNFi* Tumor necrosis factor inhibitor

^a^“Before pregnancy” refers to period from 20 weeks prior to conception until the positive pregnancy test

* *p* <0.05

**Fig. 1:**
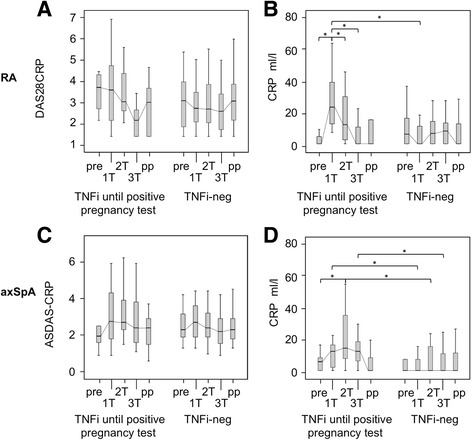
Disease activity over the course of pregnancy in patients with rheumatoid arthritis (RA) and axial spondyloarthritis (axSpA) treated with and without tumor necrosis factor inhibitors (TNFi) until early pregnancy. Disease activity is compared between patients who received TNFi until the positive pregnancy test and patients without TNFi treatment (TNFi-neg). The *upper panels* show Disease Activity Score in 28 joints based on C-reactive protein (DAS28-CRP) levels (**a**) and C-reactive protein (CRP) levels (**b**) in patients with RA (prepregnancy [pre]: number of patients [*n*] = 54, first trimester [1 T]: *n* = 59, second trimester [2 T]: *n* = 66, third trimester [3 T]: *n* = 64, postpartum [pp]: *n* = 53). The *lower panels* display Ankylosing Spondylitis Disease Activity Score based on C-reactive protein (ASDAS-CRP) levels (**c**) and CRP levels (**d**) in patients with axSpA (pre: *n* = 44, 1 T: *n* = 46, 2 T: *n* = 58, 3 T: *n* = 54, pp: *n* = 46). Box plots present the medians and the interquartile ranges. *P < 0.051

During the prepregnancy period, patients with RA treated with TNFi showed disease activity comparable to that of patients without TNFi treatment during the same period. Among the 15 patients treated with TNFi during at least 20 weeks before conception, all discontinued the treatment at the positive pregnancy test; the median time point of discontinuation was gestational week 3. After the discontinuation of TNFi, the relative risk of a flare during pregnancy was 3.33 (95% CI 1.8–6.1). Patients with RA who discontinued TNFi showed active disease at the first trimester and peaking CRP levels (*P* = 0.04 prepregnancy to first trimester, *P* = 0.03 first trimester to second trimester) (Fig. 1). The aggravation of disease activity led to a treatment adjustment in 60% of the patients. In most patients with RA without TNFi treatment, disease activity remained stable throughout pregnancy, with DAS28-CRP scores ranging from 2.6 to 3.1 and low CRP levels (Fig. 1a, b). The therapies received by patients with RA with and without TNFi are shown in Table 2. Among patients without TNFi, a higher proportion received pregnancy-compatible drugs such as sulfasalazine, hydroxychloroquine, GCs, and nonsteroidal anti-inflammatory drugs before and during pregnancy than those treated with TNFi.

Among patients with axSpA, 24.6% showed active disease with ASDAS scores greater than or equal to 2.1 before pregnancy. Flares of disease activity during pregnancy occurred in 25% of patients with axSpA and were most often seen in the second half of pregnancy (Table 2). The discontinuation of TNFi treatment at the positive pregnancy test (*P* = 0.017), elevated CRP levels (*P* = 0.006), and active disease at early pregnancy were associate with flares during pregnancy in these women (Table 3, Additional file 1: Table S1).

Among the 61 patients with axSpA, 24 patients received TNFi treatment before pregnancy and had a median ASDAS-CRP of 1.95 preconceptionally. After discontinuation of TNFi treatment at the positive pregnancy test (median time point of discontinuation was gestational week 3), the relative risk of flare was 3.08 (95% CI 1.2–7.9) (Table 3). Patients with axSpA who discontinued TNFi treatment experienced high disease activity throughout pregnancy, with ASDAS-CRP scores ranging between 2.4 and 2.8 and CRP peaking at the second trimester (Fig. 1c, d). There was no patient with more than one flare episode during pregnancy. Among patients with axSpA discontinuing TNFi, an initiation or restart of medication was required in 62.5% during gestation. In patients with axSpA without TNFi treatment, ASDAS-CRP scores ranged from 2.2 to 2.7 throughout pregnancy, and stable low CRP levels were measured. Of these patients, 10.8% required a start of new medication during pregnancy (Table 2). Comparing patients without TNFi treatment and patients discontinuing TNFi treatment at the positive pregnancy test, higher CRP values were found in the latter group throughout pregnancy (*P* = 0.04 for first trimester, *P* = 0.03 for second trimester, *P* = 0.01 for third trimester) (Fig. 1d).

### Response to initiation of TNFi or GC treatment during pregnancy

**Fig. 2:**
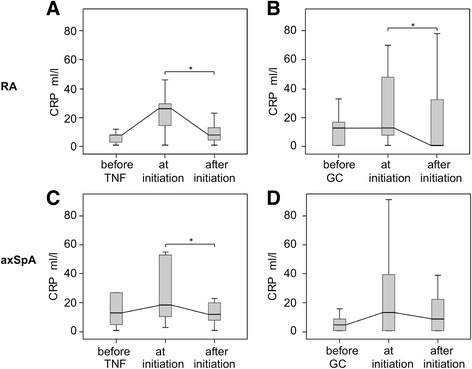
Response to treatment of flares with tumor necrosis factor inhibitor (TNFi) or glucocorticosteroid (GC) treatment in patients with rheumatoid arthritis (RA) and axial spondyloarthritis (axSpA) during pregnancy. The *upper panels* show the time course of C-reactive protein (CRP) levels in patients with RA in whom TNFi treatment (**a**) or GC treatment (**b**) was initiated during pregnancy. The *lower panels* show the time course of CRP levels in patients with axSpA in whom TNFi treatment (**c**) or GC treatment (**d**) was initiated during pregnancy. Box plots present the medians and the interquartile ranges. *P < 0.05

During pregnancy, 25 patients with RA (flare in *n* = 22, increase of disease activity in *n* = 3) and 19 patients with axSpA (flare in *n* = 15, increase of disease activity in *n* = 4) experienced a disease aggravation with the need to step up medication. Among patients with RA, 9 patients started TNFi treatment and 16 started GC treatment during pregnancy (Table 2). Upon TNFi treatment, median CRP values decreased significantly from 26 mg/L to 8 mg/L (*P* = 0.03) (Fig. 2a), and median DAS28-CRP scores decreased from 4.25 to 2.60. Upon initiation of GC treatment in pregnant patients with RA, median CRP values of 13 mg/L decreased to normal levels (*P* = 0.04) (Fig. 2b), and DAS28-CRP dropped from 3.95 to 2.45, indicating inactive disease.

Among patients with axSpA, TNFi treatment was initiated in 11 and GC treatment in 15 during pregnancy (Table 2). Upon initiation of TNFi, pregnant patients with axSpA showed a significant decrease of median CRP levels from 18.5 mg/L to 12 mg/L (*P* = 0.04) (Fig. 2c). In spite of the improved CRP levels, disease activity remained high, with slightly elevated CRP levels and median ASDAS-CRP scores of 4.0 before and 3.7 after initiation of TNFi therapy (not shown). Upon GC treatment, elevated CRP values persisted, with a median CRP level of 12 mg/L (Fig. 2d) and a median ASDAS-CRP score of 3.14.

## Discussion

For the management of patients with RA and axSpA who are planning a pregnancy, both disease activity and therapeutic regimens have to be considered with regard to maternal and fetal health. Any beneficial effect of pregnancy on diseases such as RA could buffer a reduction of the antirheumatic therapy [2, 3, 7]. However, the adjustment of therapy around conception might also be associated with disease reactivation during gestation, especially in diseases such as axSpA, in which no major beneficial effect has been observed during pregnancy [15]. In the present study, disease flares during pregnancy were observed in 29% of patients with RA and 25% of patients with axSpA. Interestingly, our study shows that, in the subgroup of patients with RA and axSpA treated with TNFi for at least 5 months before conception, the discontinuation of TNFi early in pregnancy could be a risk factor for disease flares during pregnancy. Both groups of patients experienced an aggravation of disease several weeks after withdrawal of TNFi. Disease-specific characteristics as well as disease activity at the time of TNFi withdrawal might play a role in this respect because elevated disease activity in early pregnancy is another risk factor for a flare during the course of pregnancy. In nonpregnant patients with axSpA, the discontinuation of TNFi leads to disease flares in 76% to 100% [16]. In nonpregnant patients with RA, the results vary with regard to whether the withdrawal of TNFi may lead to a deterioration of disease activity [17, 18]. Factors predictive of maintaining low disease activity in RA include low disease activity at the time of TNFi discontinuation [17]. However, others report immediate disease deterioration in patients with early RA in whom TNFi were withdrawn after they achieved remission while receiving TNFi and methotrexate [18]. Presumably, the time in remission is a prerequisite in patients with RA for persisting clinical benefits after TNFi discontinuation [17]. Accordingly, withdrawing TNFi later in pregnancy in patients with sustained inactive disease did not result in a flare in patients with inflammatory bowel disease [19].

In patients with RA without TNFi, 63.3% of whom were on pregnancy-compatible disease-modifying drugs, both the DAS28-CRP scores as well as CRP levels showed low disease activity during the course of pregnancy, and only 26.7% of patients required initiation of therapy. This confirms that patients with RA with low disease activity at the first trimester mostly continue with stable and low disease activity throughout gestation [3]. By contrast, the possible pregnancy-ameliorating effect in RA was not strong enough to compensate the disease aggravation that occurred after TNFi discontinuation around conception. In addition, the subpopulation of patients treated with TNFi in the period before pregnancy represents patients with more severe disease because symptoms were not sufficiently controlled with disease-modifying antirheumatic drugs alone. In the latter patient group, smoldering disease activity might erupt to disease flares after TNFi discontinuation even under the tolerance-inducing condition of pregnancy. Sixty-three percent of patients with axSpA who discontinued TNFi around conception had increased CRP levels at the second trimester. Disease flares in this group occurred around gestational week 20, which corresponds to the median time to flare of 16 weeks after discontinuation of TNFi in nonpregnant patients with axSpA [16]. Patients with axSpA not treated with TNFi before conception showed stable and low CRP levels but high ASDAS-CRP scores throughout gestation. The discrepancy could be explained by the composition of the ASDAS-CRP, which consists of the objective CRP as well as the subjective parameters [11]. A previous study comparing clinical scores and the Medical Outcomes Study 36-item Short Form Health Survey also confirmed the importance of subjective symptoms resulting in high disease activity measured in patients with axSpA throughout pregnancy [20].

The success of new initiation or reintroduction of therapy for the treatment of flares differed significantly in pregnant patients with RA and axSpA. In RA, the start of TNFi because of flare in pregnancy resulted in improvement and in achievement of disease remission in the majority of patients. The median CRP levels decreased by 69% after the start of therapy and normalized during the third trimester. By contrast, in patients with axSpA in whom TNFi treatment was initiated, median CRP levels decreased by only 35%, and CRP levels remained elevated during pregnancy. Thus, high disease activity in axSpA could be ameliorated but not controlled by restart or initiation of TNFi in pregnancy. Our results for patients with RA and axSpA suggest that the initiation of TNFi during pregnancy may be more effective in patients with active RA than in patients with active axSpA.

GC therapy has been accepted for treating flares of rheumatic disease during pregnancy [21]. Our study showed that the response of CRP values to GC treatment differed in patients with RA and patients with axSpA. Patients with RA responded with a significant decrease of disease activity and CRP levels after the start of GC treatment during pregnancy, whereas patients with axSpA showed a minimal decrease of CRP. This weak response in patients with axSpA is consistent with the lack of effect of GC treatment in nonpregnant patients with axSpA [22].

The strength of our study is the prospective design and the inclusion of two rheumatic diseases with different responses to pregnancy. It is the first report on the effect of discontinuing TNFi in early pregnancy, and on the response to the restart or the initiation of medication at a flare in pregnant patients with RA and axSpA. A limitation is the number of TNFi-treated patients, which did not allow more detailed statistical analysis.

## Conclusions

Elevated disease activity and TNFi discontinuation in early pregnancy may cause a relapse of disease activity in patients with RA and axSpA. Restart of medication controls disease activity in pregnant patients with RA but shows insufficient effect in pregnant patients with axSpA. The data indicate that tailored medication should be considered beyond conception to stabilize low disease activity and to prevent a flare during pregnancy.

## Additional file

Additional file 1: Table S1. Risk factors of for severe flares in RA [23] and axSpA [24] during the course of pregnancy. (DOCX 23 kb)

## Abbreviations

ASAS: Assessment of SpondyloArthritis international Society
ASDAS-CRP: Ankylosing Spondylitis Disease Activity Score based on C-reactive protein
axSpA: Axial spondyloarthritis
CRP: C-reactive protein
DAS28-CRP: Disease Activity Score in 28 joints based on C-reactive protein
DMARD: Disease-modifying antirheumatic drug
GC: Glucocorticosteroid
HCQ: Hydrochloroquine
HLA: Human leukocyte antigen
NSAID: Nonsteroidal anti-inflammatory drug
RA: Rheumatoid arthritis
RR: Relative risk
SpA: Spondyloarthritis
SSZ: Sulfasalazine
TNFi: Tumor necrosis factor inhibitor
