# Supplementary material for: Risk factors for flare and treatment of disease flares during pregnancy in rheumatoid arthritis and axial spondyloarthritis patients
Source: Arthritis Res Ther. 2017 Mar 20;19:64. doi: 10.1186/s13075-017-1269-1 (PMC5359860; doi:10.1186/s13075-017-1269-1)
Supplement: Additional file 1: Table S1. — Risk factors of for severe flares in RA [23] and axSpA [24] during the course of pregnancy. (DOCX 23 kb) [file 13075_2017_1269_MOESM1_ESM.docx]

**Table S1** Risk factors of for severe flares in RA [23] and axSpA [24] during the course of pregnancy.

|  | **RA patients *n* = 75** | | | | **axSpA patients *n* = 61** | | | |
| --- | --- | --- | --- | --- | --- | --- | --- | --- |
|  | Severe flare = increase of ≥ 0.6  and DAS28-CRP ≥ 3.2 | | | | severe flare = increase of ≥ 0.6  and ASDAS-CRP ≥ 1.3 | | | |
|  | yes | no | RR (95% CI) | p | yes | no | RR (95% CI) | p |
| *Treatment* |  |  |  |  |  |  |  |  |
| TNFi before pregnancy and discontinued at pos preg test**^+^** | 8/29 (27.6) | 7/46 (15.2) | 1.5 (0.8 – 2.7) | 0.241 | 11/26 (42.3) | 13/35 (37.1) | 1.1 (0.6 -2.0) | 0.793 |
| TNFi during 1^st^ trim | 4/29 (8.7) | 4/46 (8.7) | 1.3 (0.6 – 2.9) | 0.703 | 6/26 (23.1) | 12/35 (34.3) | 0.7 (0.3 - 1.5) | 0.404 |
| GC before pregnancy | 13/29 (44.8) | 12/46 (26.1) | 1.6 (0.9 – 2.8) | 0.132 | 3/23 (11.5) | 2/35 (5.7) | 1.5 (0.7 - 3.2) | 0.642 |
| GC during 1^st^ trim | 13/29 (44.8) | 12/46 (26.1) | 1.6 (0.9 – 2.8) | 0.132 | 5/26 (19.2) | 2/35 (5.7) | 1.8 (1.0 - 3.2) | 0.125 |
| DMARDs before pregnancy | 15/29 (51.7) | 17/46 (37.0) | 1.4 (0.8 – 2.5) | 0.238 | 2/26 (7.7) | 6/35 (17.1) | 0.6 (0.2 – 1.9) | 0.448 |
| DMARDs during 1^st^ trim | 10/29 (34.5) | 20/46 (43.5) | 0.8 (0.4 – 1.5) | 0.477 | 1/26 (3.8) | 4/35 (11.4) | 0.5 (0.1 - 2.6) | 0.382 |
| NSAIDs before pregnancy | 5/29 (17.2) | 3/46 (6.5) | 1.7 (0.9 – 3.3) | 0.248 | 7/26 (26.9) | 11/35 (31.4) | 0.9 (0.5 – 1.7) | 0.781 |
| NSAID during 1^st^ trim | 6/29 (20.7) | 3/46 (6.5) | 1.9 (1.1 – 3.4) | 0.081 | 9/26 (34.6) | 9/35 (25.7) | 1.3 (0.7 – 2.3) | 0.572 |
|  |  |  |  |  |  |  |  |  |
| *Disease activity* |  |  |  |  |  |  |  |  |
| Active disease before pregnancy^‡^ | 6/29 (20.7) | 8/46 (17.4) | 1.1 (0.6 – 2.3) | 0.767 | 4/26 (15.4) | 2/35 (5.7) | 1.7 (0.9 – 3.2) | 0.387 |
| Active disease during 1^st^ trim | 15/29 (51.7) | 3/46 (6.5) | 0.3 (0.2 – 0.5) | **0.001*** | 11/26 (42.3) | 5/35 (14.3) | 2.1 (1.2 – 3.5) | **0.019*** |
| Elevated CRP before pregnancy^‡^ | 5/29 (17.2) | 6/46 (13.0) | 0.8 (0.4 – 1.7) | 0.740 | 22/26 (84.6) | 32/35 (91.4) | 0.7 (0.3 – 1.5) | 0.446 |
| Elevated CRP during 1^st^ trim | 13/29 (44.8) | 7/46 (15.2) | 0.4 (0.3 – 0.8) | **0.007*** | 7/26 (26.9) | 6/35 (17.1) | 1.3 (0.7 – 2.5) | 0.528 |

n / total (%); before pregnancy, period from 20 weeks prior to conception until the positive pregnancy test; ^+^pos preg test, positive pregancy test; TNFi, TNF inhibitors; NSAIDs, nonsteroidal anti-inflammatory drugs used until gestational week 32; GC, glucocorticoids; DMARDs, disease-modifying antirheumatic drugs.
